# Supplementary material for: Experience and lessons learned from multi-modal internet-based recruitment of U.S. Vietnamese into research
Source: PLoS One. 2021 Aug 13;16(8):e0256074. doi: 10.1371/journal.pone.0256074 (PMC8363004; doi:10.1371/journal.pone.0256074)
Supplement: Attachment — Submitted filename: Reviewers comments.docx [file pone.0256074.s001.docx]

**Abstract**

The ABSTRACT is well written, addressing the investigated problems intelligently.

**Introduction**

The INTRODUCTION is also well-structured, highlighting the problems of drawing samples from online data collection approach eloquently. Besides, the existing literature is well-documented to justify the research questions raised by the authors.

**Materials and methods**

The reviewer really appreciates the METHODOLOGY, as the authors have already addressed the limitations of doing this research. However, the authors are requested to delete “: Survey” from the Recruitment procedures as the study is based on survey only. Furthermore, the authors are requested to specify the sampling procedure for ‘community-based organizations,’ ‘Facebook groups’ and so on, except for ‘snowball sampling’ group. The authors are advised to use a sub-heading ‘Measurement’ under which they are requested to place ‘Sociodemographic characteristics,’ ‘Acculturation-related characteristics’ and ‘Mobile health usage,’ instead of repeating Measurement for each sub-heading.

It is not clear how did the authors select the participants for interviews, how and what tools they used to interview for participants for qualitative interviews.

The authors are requested to specify the procedures of interviewing the participants for qualitative interview – whether it was face-to-face, or telephonic or other procedure of data collection maintain social distancing.

**Results**

The results are well presented and pointed out some critical issues regarding the short-falls and advantages of using internet-based recruitment of participants during the COVID-19.

**Discussion**

The reviewer also appreciates the DISCUSSION penned by the authors. Yet, the DISCUSSION lacks recent empirical evidence regarding the problems that are critical to understand the ongoing situation of recruiting participants during the COVID-19 pandemic.

**Conclusion**

Th author are requested to point out whether this study is generalizable to other countries or not, and how other nations could cope with the emerging problems of the online recruitment during the COVID-19 pandemic.

**Others**

The authors are requested to follow the PLoS ONE reference style properly. Many citations did not comply with the reference style suggested by PLoS ONE.
